# Supplementary material for: Modelling onchocerciasis-associated epilepsy and the impact of ivermectin treatment on its prevalence and incidence
Source: Nat Commun. 2024 Jul 25;15:6275. doi: 10.1038/s41467-024-50582-9 (PMC11272922; doi:10.1038/s41467-024-50582-9)
Supplement: Supplementary file 1 — Supplementary Information [file 41467_2024_50582_MOESM1_ESM.pdf]

Supplementary Information

Modelling Onchocerciasis-Associated Epilepsy and the Impact of Ivermectin Treatment on its Prevalence and Incidence

Jacob N. Stapley[1,2*], Jonathan I.D. Hamley[1,2,3,4], Martin Walker[1,2,5], Matthew A. Dixon[1,2], Robert Colebunders[6] and Maria-Gloria Basáñez[1,2*]

[1] MRC Centre for Global Infectious Disease Analysis, Department of Infectious Disease Epidemiology, School of Public Health, Imperial College London, London, UK

[2] London Centre for Neglected Tropical Disease Research, Department of Infectious Disease Epidemiology, School of Public Health, Imperial College London, London, UK

[3] Department of Visceral Surgery and Medicine, Inselspital, Bern University Hospital, University of Bern, Switzerland

[4] Multidisciplinary Center for Infectious Diseases, University of Bern, Bern, Switzerland

[5] Department of Pathobiology and Population Sciences, Royal Veterinary College, Hatfield, UK

[6] Global Health Institute, University of Antwerp, Antwerp, Belgium

*Corresponding Authors

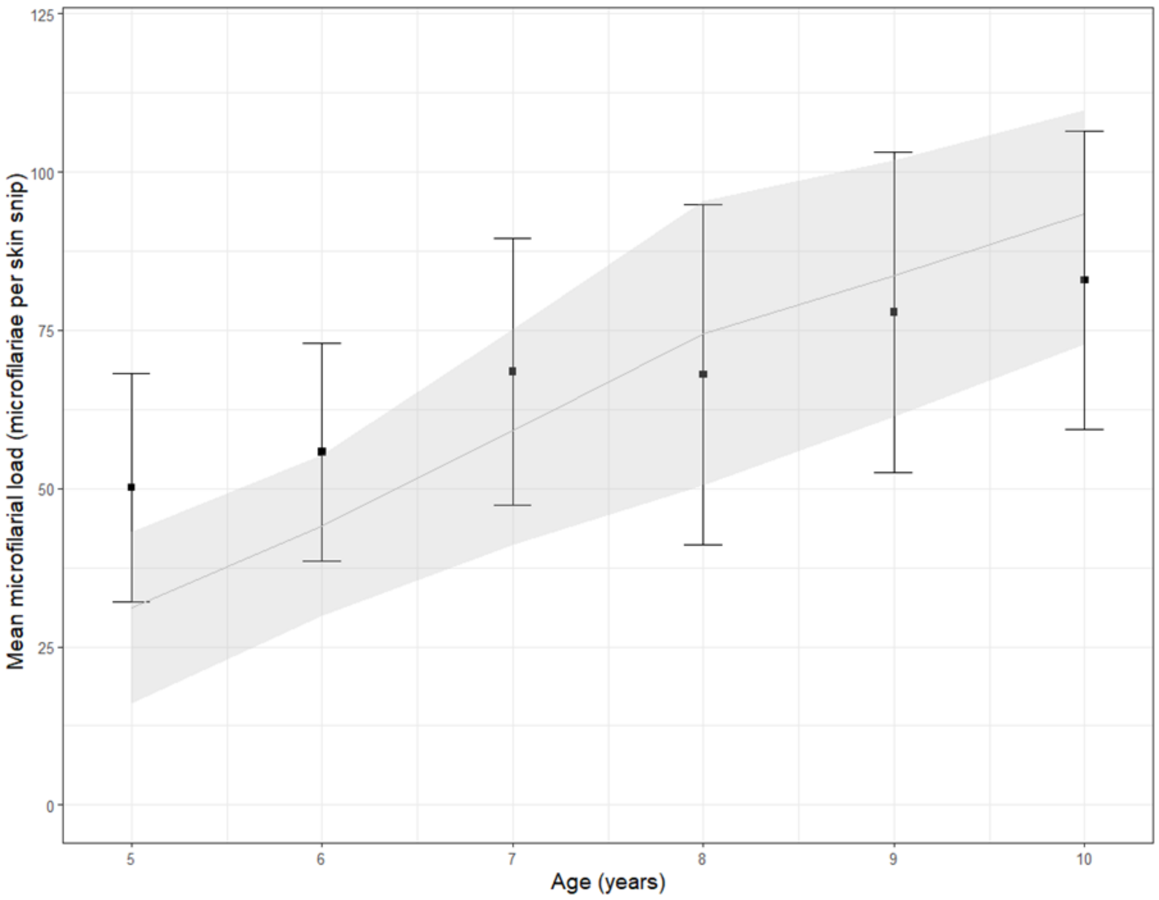

Figure S1. Observed and EPIONCHO-IBM-modelled microfilarial load (microfilariae per skin snip) of children aged 5–10 years examined in 1991–93 in the Mbam Valley, Cameroon. The squares are the data (n = 729; Table S5) from Chesnais et al. [1] and the error bars are the 95% confidence intervals around the data. The grey line represents the microfilarial load generated by EPIONCHO-IBM for ABR = 41,922 bites/person/year, estimated by fitting the model by least squares to these data. The grey shaded area illustrates uncertainty around the ABR by running the model with values of 14,000 and 100,000 bites/person/year, which are the minimum and maximum values reported by Barbazan et al. [2] in the study area in 1993–94. All simulations were conducted with 300 model repeats.

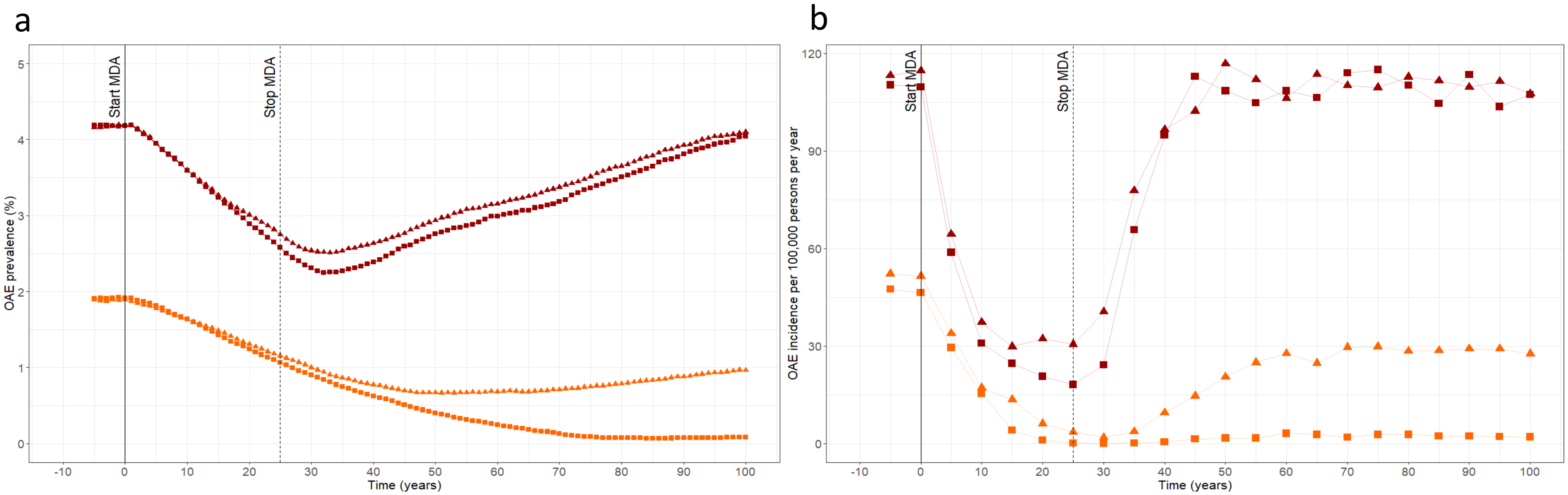

Figure S2. The modelled impact of 25 years of biannual community-directed treatment with ivermectin (CDTI) on onchocerciasis-associated epilepsy (OAE) for two levels of endemicity and therapeutic coverage. (**a**), Prevalence of OAE in the overall population (all ages). (**b**), Incidence of OAE (no. of cases/100,000 persons/year). Orange and dark red colours denote hyper- and holoendemic settings, respectively (annual biting rate (ABR) = 1,000 for 60% microfilarial (mf) prevalence; ABR = 7,300 for 80% mf prevalence). Triangles and squares represent minimal and enhanced coverage, respectively. Minimal coverage: 65% therapeutic coverage of total population and 5% systematic non-adherence (SNA); enhanced coverage: 80% therapeutic coverage of total population and 1% SNA. All simulations were conducted with 300 model repeats.

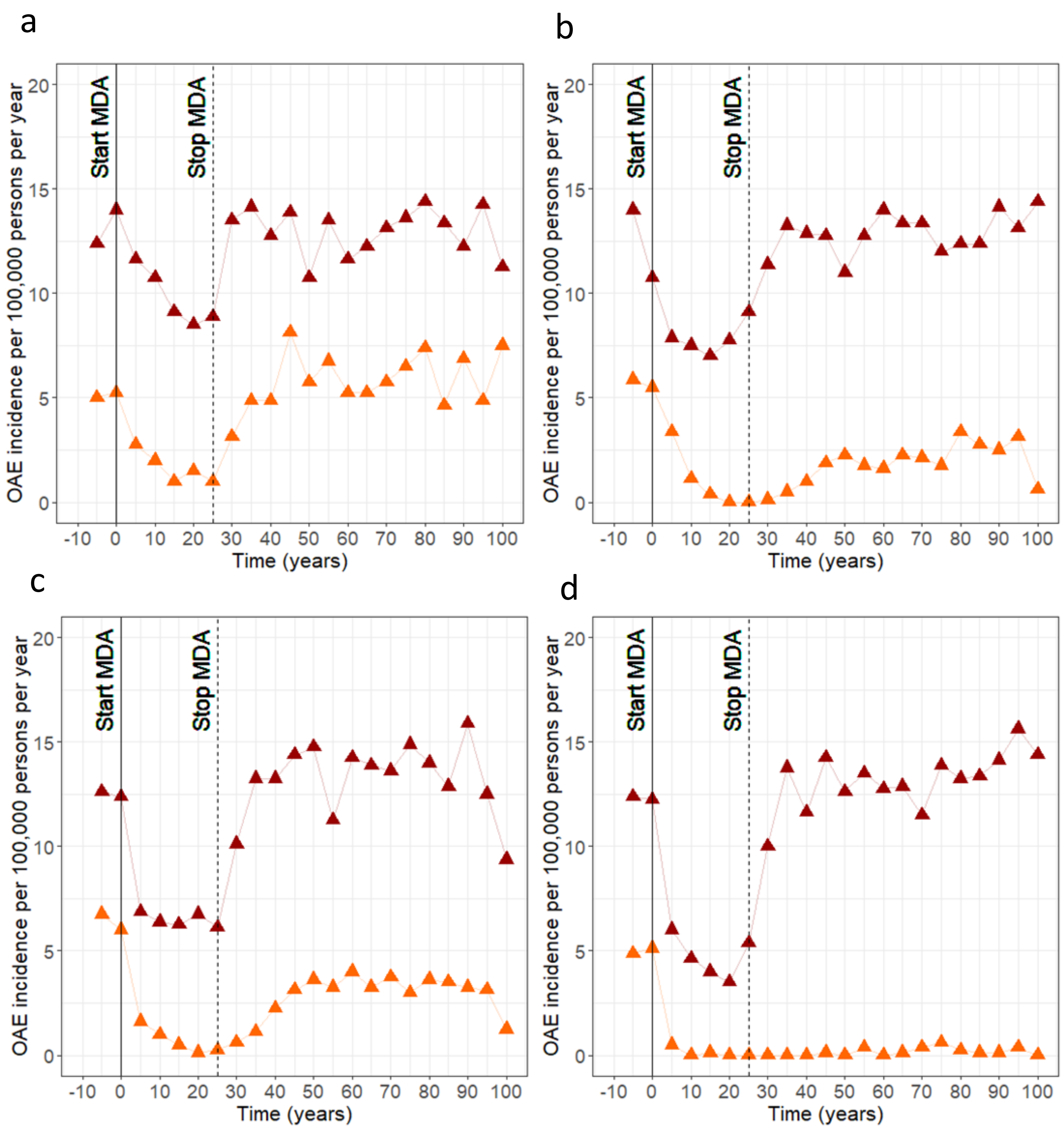

Figure S35. The modelled impact of 25 years of community-directed treatment with ivermectin (CDTI) on the contribution of untreated children (under 5 year-olds) to onchocerciasis-associated epilepsy (OAE) incidence for two levels of endemicity and therapeutic coverage. Upper panel: Annual CDTI with (**a**), minimal coverage; (**b**), enhanced coverage. Lower panel: Biannual CDTI with (**c**), minimal coverage; (**d**), enhanced coverage. Orange and dark red colours denote hyper- and holoendemicity, respectively. Annual biting rate and coverage values are as given in the legend of Figure S2. All simulations were conducted with 300 model repeats.

Table S1. Comparison of measures of central tendency and variation for microfilarial (mf) load recorded by Chesnais et al. [1] and generated by EPIONCHO-IBM (annual biting rate (ABR) = 41,992 bites/person/year, k_E = 0.3)

Age in 1991–93 (years)	Arithmetic mean (mf/skin snip)		Standard deviation (SD) (mf/skin snip)		Median (mf/skin snip)		Interval quartile range (IQR) (mf/skin snip)	
	Chesnais et al. [1]	EPIONCHO-IBM	Chesnais et al. [1]	EPIONCHO-IBM	Chesnais et al. [1]	EPIONCHO-IBM	Chesnais et al. [1]	EPIONCHO-IBM
5	50.1	31.1	93.7	16.5	15.0	29.0	0.5–58.0	12.5–45.5
6	55.8	44.1	106.7	23.8	11.0	42.0	0.0–55.0	18.2–65.8
7	68.4	59.1	130.8	28.9	13.8	56.8	0.5–72.3	27.9–85.7
8	68.0	74.5	134.0	37.9	21.5	69.0	1.0–65.5	31.1–106.9
9	77.8	83.6	135.3	37.0	21.0	82.3	2.0–88.5	45.3–119.3
10	82.9	93.3	134.2	44.5	16.5	89.2	1.5–96.5	44.7–133.7

Table S2. Incidences of onchocerciasis-associated epilepsy (OAE) generated by EPIONCHO-IBM compared to values in [1]. Incidence as number of cases of epilepsy in 100,000 person-years in the modelled population for children aged 5–10 years examined in 1991–93 who were followed up in 2017 when they were aged 30–35 years, after 19 years of annual community-directed treatment with ivermectin (CDTI). Simulation of CDTI followed the therapeutic coverage values presented in Kamga et al. [3]. The values presented are the average of 400 model runs. The methodology used to calculate values of incidence per 100,000 person-years (PY) followed that of Chesnais et al. [1]

Age (a) in 1991–93 (years)	Number of children of age a in the modelled population (400 individuals)	Person-years (PY) in modelled population	Cases of epilepsy in modelled population	Modelled incidence (cases per 100,000 PY) EPIONCHO-IBM	Recorded incidence (cases per 100,000 PY) (95% CI) [1]
5	5.42	128.34	0.37	290.25	530 (310–920)
6	5.31	125.85	0.39	309.90	280 (150–510)
7	5.30	125.61	0.43	342.33	400 (240–680)
8	5.11	120.99	0.41	334.74	370 (180–730)
9	5.06	119.80	0.39	325.53	270 (130–560)
10	4.93	116.84	0.35	299.55	270 (130–540)
**Total**	**31.12**	**737.54**	**2.34**	**317.27**	**350 (270–450)**

Text S1. EPIONCHO-IBM

EPIONCHO-IBM is a stochastic, individual-based model developed from its deterministic, population-based (EPIONCHO) predecessors [4–6]. The model tracks, in a closed population of 400 individuals, the number of adult (male and female) *O. volvulus* worms in human hosts, the number of microfilariae in their skin, and the number of infective, L3 larvae in blackfly vectors. Parasite population abundance is regulated in humans and flies by density-dependent processes operating upon establishment of incoming worms within humans; establishment of L3 larvae within vectors, and vector survival [5,7]. Excess mortality of humans as a function of their mf load [8,9] has not yet been included. For sub-Saharan Africa settings, the model has been parameterised for savannah *O. volvulus–S. damnosum* sensu lato [4–7]. The baseline (pre-control) mf prevalence (endemicity level) is determined by the annual biting rate (ABR, no. bites/person/year) [6,7].

Individuals within the model are differentially exposed to blackfly bites depending on their age and sex [4] (Figure S4, reproduced from Hamley et al. [10]) as well as on their individual-specific exposure, $E_{(i)}$. This individual exposure factor is assigned at birth and drawn from a gamma distribution,

$$E_{(i)} \sim G(k_E, \beta_E) \qquad \text{(S1)}$$

where k_E and β_E are the shape and rate parameters, respectively. It is assumed that k_E = β_E, such that the mean exposure in the population is unity, i.e., blackfly bites are distributed among hosts with an average exposure given by the ABR [7]. Values of k_E (ranging from 0.2 to 0.4) are accompanied by specific sets of density dependence parameters such that lower values of k_E (indicating stronger overdispersion in exposure heterogeneity among hosts) correspond to stronger density dependence in parasite establishment within humans and vice versa, as estimated from fitting EPIONCHO-IBM to data on mf prevalence and mf load as a function of ABR [7] (Table S3). Therefore, for a given ABR and k_E, EPIONCHO-IBM generates the adult worm burden and mf load of each individual host in the population, also allowing for calculation of mf prevalence.

Mass ivermectin treatment-based interventions are modelled by incorporating the temporal dynamics following treatment of the microfilaricidal and embryostatic effects of ivermectin with parameter values as estimated in Basáñez et al. [11]. A permanent sterilising effect on

adult worms is also included [12]. The modelling of therapeutic coverage (proportion of individuals receiving ivermectin at each treatment round in the total population) and systematic non-adherence (proportion of eligible individuals never receiving treatment) are modelled as previously described, with the value of therapeutic coverage referring to the mean treatment probability in any treatment round [7,10]. A complete description of EPIONCHO-IBM is presented in the Supplementary File of Hamley et al. [7], and the R code can be found at: https://github.com/mrc-ide/EPIONCHO.IBM.

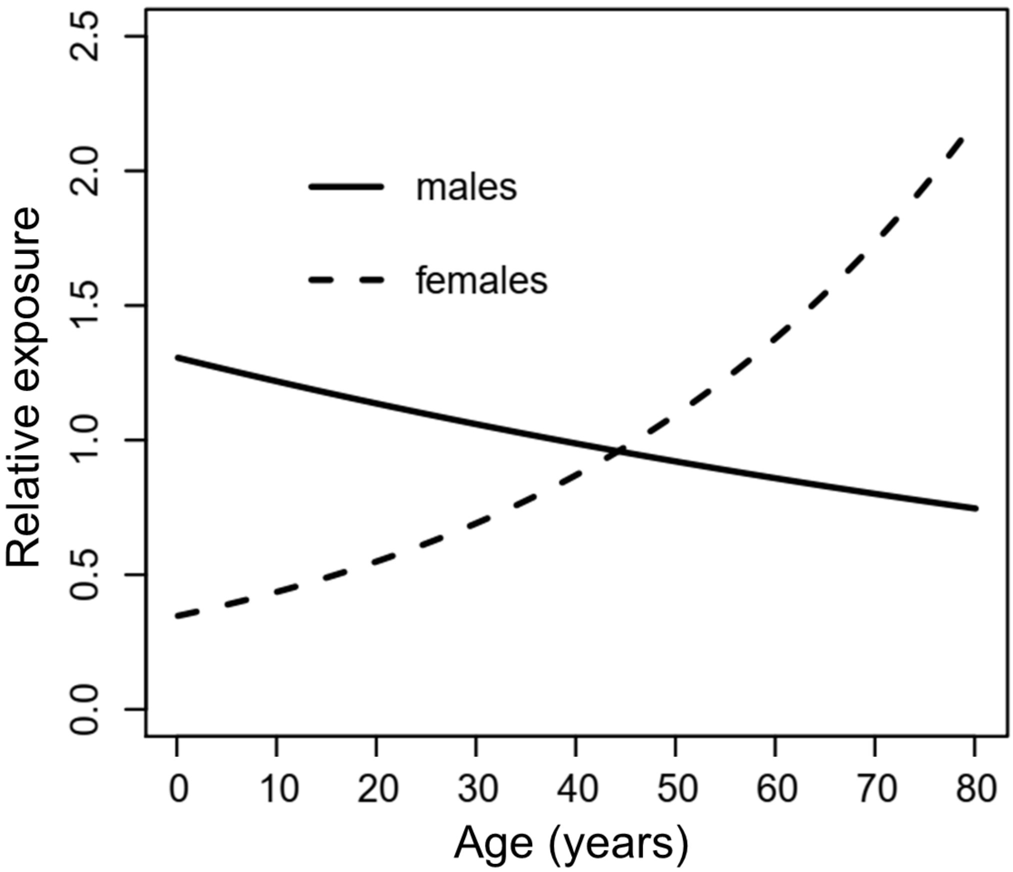

Figure S4. Age- and sex-dependent exposure function in EPIONCHO-IBM as inferred by fitting the model described in [4] to age- and sex-specific profiles of mf load from Cameroon. Figure reproduced from Hamley et al. 2020 [10] under terms of Creative Commons CC BY license.

Table S3. Density dependence parameters determining parasite establishment within humans as a function of the annual transmission potential (ATP) for different values of parameter $\boldsymbol{k_E}$ of the gamma distribution describing inter-individual exposure heterogeneity (from [7])

Shape parameter of gamma distribution	Density dependence parameters for parasite establishment within humans		
k_E	δ_{H_0}	δ_{H_∞}	c_H
0.2	0.385	0.003	0.008
0.3	0.186	0.003	0.005
0.4	0.118	0.002	0.004

δ_{H_0} is the proportion of L3 larvae developing to adult worms within the human host, per bite, when ATP tends to 0; δ_{H_∞} is the proportion of L3 larvae developing to adult worms within the human host, per bite, when ATP is very large, and c_H is the severity of transmission intensity-dependent parasite establishment within humans.

Text S2. Epidemiological and Entomological Data

The retrospective study of Chesnais et al. [1] was conducted in 7 of the original 25 villages surveyed for *Onchocerca volvulus* parasitological data in the Mbam Valley, Central Cameroon, in 1991–93 as described in the Main Text. Figure S5 shows the locations of villages in the Mbam Valley studied during both the initial (1991–93) and the follow-up (2017) epilepsy surveys [1]. This is a forest–savannah mosaic area, where the vectors belong to the *Simulium damnosum* sensu lato (s.l.) species complex [13]. During the initial epidemiological study, the region had not received ivermectin treatment, which started in 1998 [1]. According to the study by Pion et al. [14], the microfilarial (mf) prevalence in the villages, in 1991–93, ranged from 80.9% (95% CI=75.9–85.0%) to 96.8% (95% CI=93.9–98.3%), and the community microfilarial load (CMFL, the geometric mean number of mf per skin snip in those aged ≥20 years [15]) from 13.9 to 136.2 mf/ss, indicating baseline holoendemicity, with a mean mf prevalence of 86.7% and a mean CMFL of 50.6 mf/ss (Table 1 of Main Text).

Barbazan et al. [2] conducted an entomological study in the area prior to the implementation of ivermectin treatment, and reported annual biting rate (ABR) values ranging from 43,273 to 98,028 bites/person/year at Ngoro, to the northeast of the Chesnais et al. study area), and from

13,647 to 48,525 bites/person/ year at Bokito, to the southwest). Figure S5 indicates the locations of Ngoro and Bokito. The corresponding annual transmission potential (ATP) values ranged from 184 to 3,113 L3/person/year, and from 11 to 813, respectively. Overall, the mean ABR was 39,765 bites/person/year (SD = 25,991), and mean ATP of 769 (SD = 1,050) L3/person/year. The mean ATP at Ngoro was 1,583 L3/person/year. The mean number of L3/fly (0.0398) was calculated dividing the ATP at Ngoro by the overall ABR (1,583/39,765). In Ngoro, the number of L3/fly ranged from 0.0042 to 0.0426. These values were used to validate the EPIONCHO-IBM model outputs.

Data from Chesnais et al. [1] consisted of follow-up epilepsy data (collected in 2017) from 729 individuals representing 85% of the original 856 who had been examined for their mf load ~25 years previously (when aged between 5−10 years), in 1991−93. A total of 564 (77%) individuals were mf-positive and were determined to have had a median mf load of 28.5 (interquartile range (IQR): 7.8−95.5) mf/ss. The ages and mf load in 1991−93 of the 729 individuals followed up, and the proportion (by age and by binned mean mf load) suspected of having developed epilepsy during the intervening ~25-year period are shown in Table S4 and Table S5, respectively.

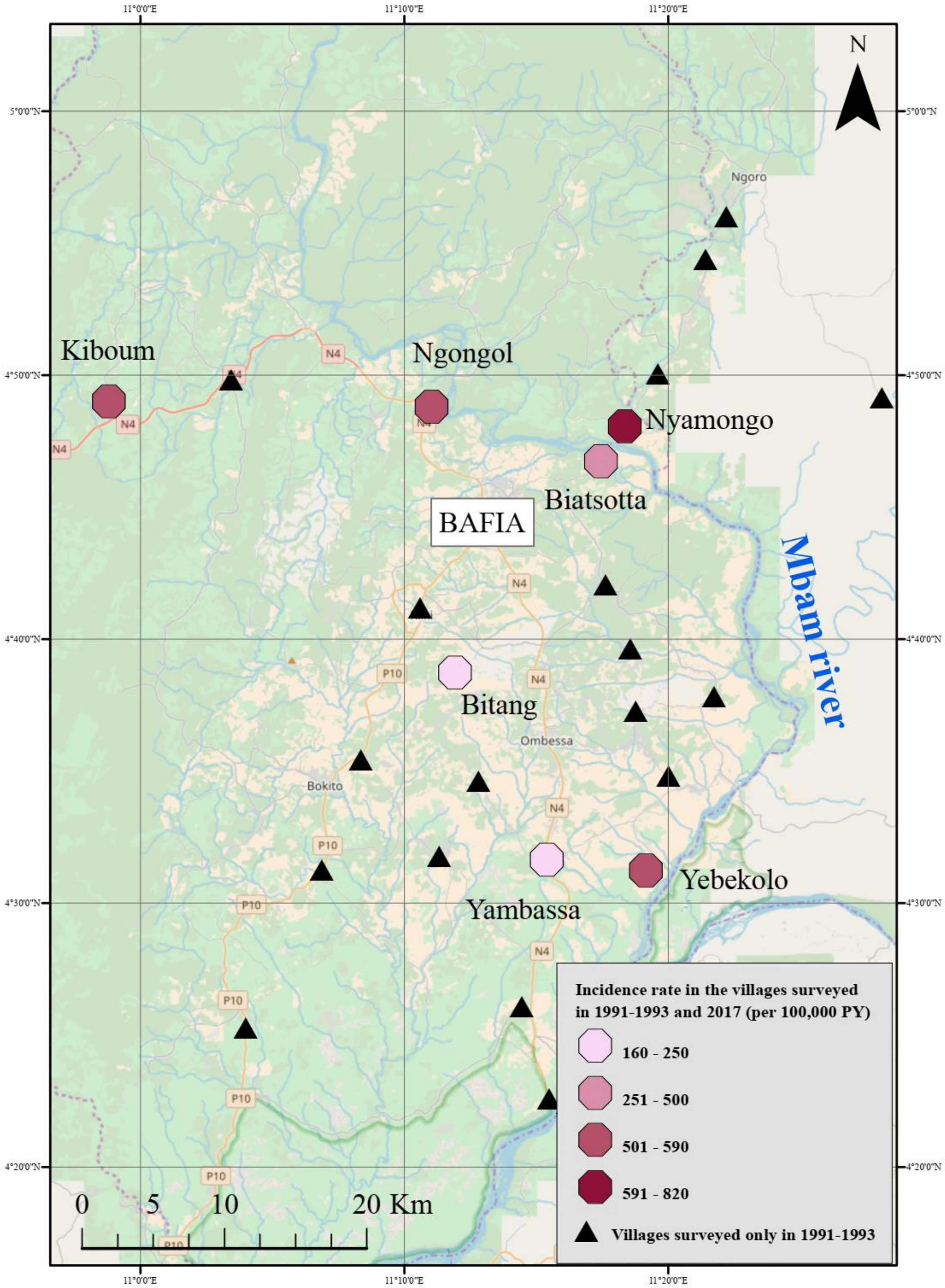

Figure S5. Location of the villages in the Chesnais et al. first retrospective cohort study [1]. The map indicates the locations of villages in the Mbam Valley visited during both the initial (1991–93) and follow-up (2017) surveys, along with the incidence rates for the 7 study of villages in 2017. Entomological studies were conducted in Ngoro and Bokito [2]. Image reprinted from The Lancet Infectious Diseases, 18, Chesnais CB, Nana-Djeunga HC, Njamnshi AK, et al., The temporal relationship between onchocerciasis and epilepsy: a population-based cohort study, 1278–86, Copyright (2018), with permission from Elsevier.

Table S4. Arithmetic mean *Onchocerca volvulus* microfilarial (mf) load in the 5–10-year old children examined in 1991–93 who were followed up by Chesnais et al. [1] in 2017 and their prevalence of epilepsy when they were aged 30–35 years

***O. volvulus* infection intensity in 1991–93**			**Epilepsy 25 years later**	
Age (years)	No. examined	Mean mf load (mf/skin snip)	No. cases	Prevalence (%) [95% CI]
5	103	50.1	13	12.6 [7.5 – 20.4]
6	148	55.8	10	6.8 [3.7 – 12.0]
7	148	68.4	14	9.5 [5.7 – 15.3]
8	95	68.0	8	8.4 [4.3 – 15.8]
9	110	77.8	7	6.4 [3.1 – 12.6]
10	125	82.9	8	6.4 [3.3 – 12.1]
Total	729	50.1	60	8.2 [6.5 – 10.5]

Table S5. Binned *Onchocerca volvulus* mean microfilarial (mf) load in the 5–10-year old children examined in 1991–93 who were followed up for epilepsy by Chesnais et al. [1] in 2017 when they were aged 30–35 years and prevalence of epilepsy by mf load category

***O. volvulus* infection intensity in 1991–93**		**Epilepsy 25 years later**	
Binned mf load*	No. examined	No. cases	Prevalence (%) [95% CI]
0	165	1	0.6 [0.1 – 3.4]
1–5	114	5	4.4 [1.9 – 9.9]
6–20	125	9	7.2 [3.8 – 13.1]
21–50	106	9	8.5 [4.5 – 15.4]
51 – 100	82	11	13.4 [7.7 – 22.5]
101 – 200	52	8	15.4 [8.0 – 27.5]
>200	85	17	20.0 [12.9 – 29.7]
Total	729	60	8.2 [6.5 – 10.5]

*For each participant 2 skin snips were taken (each from the right and left iliac crests) and incubated for 24 h in saline to enumerate the emerged microfilariae. The arithmetic mean number of microfilariae per skin snip for each individual was calculated. These means were binned into 7 mf load categories.

Text S3. Dose-response relationship between *Onchocerca volvulus* mf load and probability of developing epilepsy

The retrospective cohort studies by Chesnais et al. [1,16] provide the strongest evidence to date of temporality (one of the Bradford-Hill criteria for causation [17]) in the relationship between past mf load and developing epilepsy. These data reveal a relationship between *O. volvulus* mf load in children (aged 5–10 [1] or 5–15 years [16]) during the initial sampling and the risk of developing epilepsy later in life as measured in the follow-up studies. The higher the mf load in childhood, the greater the probability of having developed epilepsy (see Table 1 of Chesnais et al. [1]). Therefore, we use the term of onchocerciasis-associated epilepsy (OAE). The narrow age-window (3–18 years) associated with the onset of OAE [18] suggests that its occurrence is not the result of a gradual mf accumulation over a lifetime, but rather of an 'acute event', a rapid increase in mf load during childhood as a result of high cumulative exposure. This would, alongside possible genetic factors, explain why not every individual with a high mf load develops OAE [1]. Chesnais et al. documented that a high mf load at age 5 years had a more consequential impact than at age 10 or 15 years [1,16]. This, together with other work [18], indicates that beyond the age of 18 years, the chance of OAE onset is near zero. However, we did not expand the age-range to 3–18 years for OAE onset in our model given the lack of mf data beyond 15 years of age in [1,16]. Therefore, those aged 3–15 years are those who contribute to OAE in our model (see Main Text).

We made the following assumptions to model the relationship between *O. volvulus* mf load and OAE:

(1) The sampling performed by Chesnais et al. [1] was representative and non-biased, with all suspected cases of epilepsy being OAE.

(2) Each individual within our sampling age range (3–15 years) was assumed to be equally susceptible to developing OAE.

(3) New-born individuals became susceptible to developing OAE at a single randomly assigned age within our sampling range (3–15 years). When this age is reached, if an individual harbours at least a pair of male and female worms, they become OAE-positive according to a (single) Bernoulli trial with probability $Y(M_{(i)})$,

$$Y(M_{(i)}) = \exp\{\beta_1[\log(M_{(i)} + 1)] + \beta_0\} \quad \text{(S2)}$$

where $Y(M_{(i)})$ represents OAE onset probability as a function of mf load, $M_{(i)}$ is the individual mf load (taken as the mid-point of the binned mean mf load in Table S5), β_0 is the intercept, such that a mf load of zero can be associated with a non-zero OAE onset probability (to account for sampling error in the skin-snipping process), and β_1 is the strength of the association between mf load and the probability of developing OAE. The function in Eqn. S2 was fitted to the data in Table S5 using a generalised linear model (GLM) with a log link using R v. 4.3.2 (https://cran.r-project.org/bin/windows/base/) [19].

(4) Once individuals become positive for OAE, they remain positive for life, and we assume that they are not at increased risk of mortality (but see [20]).

(5) Simulated populations were closed, with no loss to follow-up or migration.

Text S4. Modelling for policy: PRIME-NTD

For the analyses presented, we adhered to the Five Principles of the Neglected Tropical Disease (NTD) Modelling Consortium for good practice in policy-relevant NTD modelling [21]. Table S6 briefly describes the five tenets, how they were fulfilled, and where in the Main Text and/or Supplementary Information they can be found.

Table S6. Policy-Relevant Items for Reporting Models in Epidemiology of Neglected Tropical Diseases (PRIME-NTD) summary table [21].

Principle	What has been done to satisfy the principle?	Where in the manuscript is this described?
**Stakeholder engagement**	Discussions with a range of modelling and policy-focused collaborators, as well as epilepsy experts	Author list, Acknowledgements section
**Complete model documentation**	References to the full description of EPIONCHO-IBM are provided. An Open Access link to the code has been given	Methods section, Supplementary Information and Data accessibility section
**Complete description of data used**	The data used had been published and are described in the manuscript and cited references	Main text, Supplementary Information, Reference lists of Main Text and Supplementary Information
**Communicating uncertainty**	Sensitivity and Uncertainty analyses were conducted to investigate the influence of varying the annual biting rate (ABR) and the degree of inter-individual exposure heterogeneity upon projected onchocerciasis-associated epilepsy (OAE) prevalence. Ninety-five percent confidence intervals (or ranges, or standard deviation (SD) values) around data were presented, as well as around the fitted microfilarial (mf) load-OAE onset probability function	Methods and Results sections. Figures and figure legends. Supplementary Information figures and tables
**Testable model outcomes**	Model outcomes were tested against epilepsy prevalence and incidence in 30–35 year olds as well as against	Results and Discussion sections

	epilepsy proportional reductions arising from prolonged annual ivermectin treatment having simulated intervention history and varied ABR. In areas where biannual ivermectin mass drug administration (MDA) has been deployed to reduce OAE (e.g. hyperendemic areas of Tanzania and South Sudan), model-derived reductions in OAE prevalence and incidence could be compared with observations	

Supplementary References

1. Chesnais CB, Nana-Djeunga HC, Njamnshi AK, et al. The temporal relationship between onchocerciasis and epilepsy: a population-based cohort study. Lancet Infect Dis **2018;** 18:1278–86.
2. Barbazan P, Escaffre H, Mbentengam R, Boussinesq M. Etude entomologique sur la transmission de l'onchocercose dans une zone de transition forêt-savane du Cameroun. Bull Soc Pathol Exot **1998;** 91:178–82.
3. Kamga GR, Dissak-Delon FN, Nana-Djeunga HC, et al. Still mesoendemic onchocerciasis in two Cameroonian community-directed treatment with ivermectin projects despite more than 15 years of mass treatment. Parasit Vectors **2016;** 9:581.
4. Filipe JAN, Boussinesq M, Renz A, et al. Human infection patterns and heterogeneous exposure in river blindness. Proc Natl Acad Sci U S A **2005;** 102: 15265–70.
5. Basáñez MG, Walker M, Turner HC, Coffeng LE, de Vlas SJ, Stolk WA. River blindness: mathematical models for control and elimination. Adv Parasitol **2016;** 94:247–341.
6. Walker M, Stolk WA, Dixon MA, et al. Modelling the elimination of river blindness using long-term epidemiological and programmatic data from Mali and Senegal. Epidemics **2017;** 18:4–15.
7. Hamley JID, Milton P, Walker M, Basáñez MG. Modelling exposure heterogeneity and density dependence in onchocerciasis using a novel individual-based transmission model, EPIONCHO-IBM: implications for elimination and data needs. PLoS Negl Trop Dis **2019;** 13:e0007557.
8. Little MP, Breitling LP, Basáñez MG, Alley ES, Boatin BA. Association between microfilarial load and excess mortality in onchocerciasis: an epidemiological study. Lancet **2004;** 363:1514–21.
9. Walker M, Little MP, Wagner KS, Soumbey-Alley EW, Boatin BA, Basáñez MG. Density-dependent mortality of the human host in onchocerciasis: relationships between microfilarial load and excess mortality. PLoS Negl Trop Dis **2012;** 6:e1578.
10. Hamley JID, Walker M, Coffeng LE, et al. Structural uncertainty in onchocerciasis transmission models influences the estimation of elimination thresholds and selection of age groups for seromonitoring. J Infect Dis **2020;** 221(Suppl 5):S510–S518.
11. Basáñez MG, Pion SDS, Boakes E, Filipe JAN, Churcher TS, Boussinesq M. Effect of single-dose ivermectin on *Onchocerca volvulus*: a systematic review and meta-analysis. Lancet Infect Dis **2008;** 8:310–22.
12. Plaisier AP, Alley ES, Boatin BA, et al. Irreversible effects of ivermectin on adult parasites in onchocerciasis patients in the Onchocerciasis Control Programme in West Africa. J Infect Dis **1995;** 172:204–10.
13. Hendy A, Krit M, Pfarr K, et al. *Onchocerca volvulus* transmission in the Mbam valley of Cameroon following 16 years of annual community-directed treatment with ivermectin,

and the description of a new cytotype of *Simulium squamosum*. Parasit Vectors **2021;** 14:563.

14. Pion SDS, Clément MCA, Boussinesq M. Impact of four years of large-scale ivermectin treatment with low therapeutic coverage on the transmission of *Onchocerca volvulus* in the Mbam valley focus, central Cameroon. Trans R Soc Trop Med Hyg **2004;** 98:520–28.
15. Remme J, Ba O, Dadzie KY, Karam M. A force-of-infection model for onchocerciasis and its applications in the epidemiological evaluation of the Onchocerciasis Control Programme in the Volta River basin area. Bull World Health Organ **1986;** 64:667–81.
16. Chesnais CB, Bizet C, Campillo JT, et al. A second population-based cohort study in Cameroon confirms the temporal relationship between onchocerciasis and epilepsy. Open Forum Infect Dis **2020;** 7:ofaa206.
17. Colebunders R, Njamnshi AK, Menon S, et al. *Onchocerca volvulus* and epilepsy: a comprehensive review using the Bradford Hill criteria for causation. PLoS Negl Trop Dis **2021;** 15:e0008965.
18. Van Cutsem G, Siewe Fodjo JN, Dekker MCJ, Amaral LJ, Njamnshi AK, Colebunders R. Case definitions for onchocerciasis-associated epilepsy and nodding syndrome: A focused review. Seizure **2023;** 107:132–35.
19. R Core Team. R: A language and environment for statistical computing. **2023;** R Foundation for Statistical Computing, Vienna, Austria. Available at: https://www.R-project.org/.
20. Bhattacharyya S, Vinkeles Melchers NVS, Siewe Fodjo JN, et al. Onchocerciasis-associated epilepsy in Maridi, South Sudan: modelling and exploring the impact of control measures against river blindness. PLoS Negl Trop Dis **2023;** 17:e0011320.
21. Behrend MR, Basáñez MG, Hamley JID, et al. NTD Modelling Consortium. Modelling for policy: the five principles of the Neglected Tropical Diseases Modelling Consortium. PLoS Negl Trop Dis **2020;** 14:e0008033.
