# Supplementary material for: Modelling onchocerciasis-associated epilepsy and the impact of ivermectin treatment on its prevalence and incidence
Source: Nat Commun. 2024 Jul 25;15:6275. doi: 10.1038/s41467-024-50582-9 (PMC11272922; doi:10.1038/s41467-024-50582-9)
Supplement: Supplementary file 2 — Reporting Summary [file 41467_2024_50582_MOESM2_ESM.pdf]

nature portfolio

Corresponding author(s): Prof. Maria-Gloria Basanez

Last updated by author(s): Jul 11, 2024

Reporting Summary

Nature Portfolio wishes to improve the reproducibility of the work that we publish. This form provides structure for consistency and transparency in reporting. For further information on Nature Portfolio policies, see our Editorial Policies and the Editorial Policy Checklist.

Statistics

For all statistical analyses, confirm that the following items are present in the figure legend, table legend, main text, or Methods section.

n/a	Confirmed	
☐	☒	The exact sample size (*n*) for each experimental group/condition, given as a discrete number and unit of measurement
☐	☒	A statement on whether measurements were taken from distinct samples or whether the same sample was measured repeatedly
☒	☐	The statistical test(s) used AND whether they are one- or two-sided<br>*Only common tests should be described solely by name; describe more complex techniques in the Methods section.*
☒	☐	A description of all covariates tested
☒	☐	A description of any assumptions or corrections, such as tests of normality and adjustment for multiple comparisons
☐	☒	A full description of the statistical parameters including central tendency (e.g. means) or other basic estimates (e.g. regression coefficient) AND variation (e.g. standard deviation) or associated estimates of uncertainty (e.g. confidence intervals)
☒	☐	For null hypothesis testing, the test statistic (e.g. *F*, *t*, *r*) with confidence intervals, effect sizes, degrees of freedom and *P* value noted<br>*Give P values as exact values whenever suitable.*
☒	☐	For Bayesian analysis, information on the choice of priors and Markov chain Monte Carlo settings
☒	☐	For hierarchical and complex designs, identification of the appropriate level for tests and full reporting of outcomes
☒	☐	Estimates of effect sizes (e.g. Cohen's *d*, Pearson's *r*), indicating how they were calculated

Our web collection on statistics for biologists contains articles on many of the points above.

Software and code

Policy information about availability of computer code

Data collection	No software was used, all data used were from published sources.
Data analysis	All analyses were undertaken using R, version 4.3.2, visualised using ggplot2 package, version 3.5.1. Model code can be found at: https://github.com/mrc-ide/EPIONCHO.IBM

For manuscripts utilizing custom algorithms or software that are central to the research but not yet described in published literature, software must be made available to editors and reviewers. We strongly encourage code deposition in a community repository (e.g. GitHub). See the Nature Portfolio guidelines for submitting code & software for further information.

Data

Policy information about availability of data

All manuscripts must include a data availability statement. This statement should provide the following information, where applicable:

- Accession codes, unique identifiers, or web links for publicly available datasets
- A description of any restrictions on data availability
- For clinical datasets or third party data, please ensure that the statement adheres to our policy

All information used for the analyses is contained in the figures, tables, and supplementary material. For this study, EPIONCHO-IBM was parameterised using previously published, publicly available data from Chesnais et al. 2018 (doi: 10.1016/S1473-3099(18)30425-0).

Research involving human participants, their data, or biological material

Policy information about studies with human participants or human data. See also policy information about sex, gender (identity/presentation), and sexual orientation and race, ethnicity and racism.

Reporting on sex and gender	We did not collect primary data on human participants. However, our EPIONCHO-IBM model is age- and sex-structured and therefore we report model outcomes separately for males and females where appropriate (Figure 3, main text). We also report the prevalence of OAE in the population aged 30-35 years disaggregated by sex, in order to compare with the results reported in Chesnais et al., 2018, Lancet Inf. Dis.
Reporting on race, ethnicity, or other socially relevant groupings	For our paper, race and/or ethnicity were not relevant. All individuals within the 3-15 year old category, which we used to simulate the onset of OAE, were assumed to be equally susceptible.
Population characteristics	Our model population is a simulated population of 400 individuals with a balanced sex ratio. We report model outputs for different age categories as appropriate. For instance, for all ages, for the relationship between OAE prevalence and vector annual biting rate; for the 30-35 year olds to compare with the study of Chesnais et al., 2018, for all simulated population above 5 years of age for the prevalence of OAE and for the under 5's to understand their contribution to OAE incidence. As above, we considered in the modelled population, all those aged 3-15 years were equally susceptible, therefore we did not include potential heterogeneity due to genetic differences.
Recruitment	We did not collect any primary data on human participants but we assumed that all epilepsy cases were as a result of OAE and we have indicated this in our paper.
Ethics oversight	Ethical approval was not necessary as this is an in-silico study which did not obtain any biological material from human participants

Note that full information on the approval of the study protocol must also be provided in the manuscript.

Field-specific reporting

Please select the one below that is the best fit for your research. If you are not sure, read the appropriate sections before making your selection.

☒ Life sciences ☐ Behavioural & social sciences ☐ Ecological, evolutionary & environmental sciences

For a reference copy of the document with all sections, see nature.com/documents/nr-reporting-summary-flat.pdf

Life sciences study design

All studies must disclose on these points even when the disclosure is negative.

Sample size	We used a modelled population of 400 individuals as it has been done in other applications of the EPIONCHO-IBM model. This population was chosen to harmonise assumptions made in other model frameworks and reflect typical sample sizes of rural African populations, endemic for onchocerciasis.
Data exclusions	We have reported all the results relevant to our research.
Replication	The model developed in this work to integrate OAE within EPIONCHO-IBM has been used to generate measures of morbidity (epilepsy) for other applications within our research group, in collaboration with the NTD MC and the Institute of Health Metrics and Evaluation. The results were fully reproducible when the model was run using R or Python.
Randomization	Randomisation is not relevant to our study, however, our model is a stochastic model, such that, each model run is different and we take the mean across 300 simulation repeats.
Blinding	Blinding is not relevant to our study as this is a modelled population

Reporting for specific materials, systems and methods

We require information from authors about some types of materials, experimental systems and methods used in many studies. Here, indicate whether each material, system or method listed is relevant to your study. If you are not sure if a list item applies to your research, read the appropriate section before selecting a response.

Materials & experimental systems

n/a	Involved in the study
☒	☐ Antibodies
☒	☐ Eukaryotic cell lines
☒	☐ Palaeontology and archaeology
☒	☐ Animals and other organisms
☒	☐ Clinical data
☒	☐ Dual use research of concern
☒	☐ Plants

Methods

n/a	Involved in the study
☒	☐ ChIP-seq
☒	☐ Flow cytometry
☒	☐ MRI-based neuroimaging

Plants

Seed stocks

Report on the source of all seed stocks or other plant material used. If applicable, state the seed stock centre and catalogue number. If plant specimens were collected from the field, describe the collection location, date and sampling procedures.

Novel plant genotypes

Describe the methods by which all novel plant genotypes were produced. This includes those generated by transgenic approaches, gene editing, chemical/radiation-based mutagenesis and hybridization. For transgenic lines, describe the transformation method, the number of independent lines analyzed and the generation upon which experiments were performed. For gene-edited lines, describe the editor used, the endogenous sequence targeted for editing, the targeting guide RNA sequence (if applicable) and how the editor was applied.

Authentication

Describe any authentication procedures for each seed stock used or novel genotype generated. Describe any experiments used to assess the effect of a mutation and, where applicable, how potential secondary effects (e.g. second site T-DNA insertions, mosiacism, off-target gene editing) were examined.
